# Among the Dentists

**Published:** 1870-10

**Authors:** 


*Among the Dentists.*—The following amusing account of a Reporter's visit to the American Dental Association meeting, held in Nashville, is taken from the "*Banner*" of that city:

"A newspaper man is supposed to feel an interest in everything, big babies, big turnips, horse races, base ball clubs, robberies, elections for constable, and all sorts of things which engage the attention and excite the interest of one or many. So we went up to mingle with the tooth pullers. A very intelligent body of men they looked to be. The majority fully up to or above the medium size, and most of them possessing serenely joyful countenances acquired from the constant contemplation of excruciating human suffering. There were a few, however, fragile and effeminate looking delegates, who, from appearances, would not be able to hold a two pound weight at arm's length. It won't do, however, to calculate on a dentist's strength from his appearance. We once knew a girlish-looking member of the profession whose complexion vied with the best pearl enamel or lilly white, whose eyes were as blue as the sky and who had nothing manly in his appearance, save his moustache. This sign of virility was coal black, and would have done honor to any man six foot by three. It was not unlike two gigantic caterpillars bristling for combat under his nose. The person who counted on his being weak labored under a painful delusion. Weak, did I say? Why, the fellow actually practiced with a pair of fifty-pound dumb-bells, and rung the changes in them with an ease and grace which wonld have done honor to a circus athlete. His muscles were like bands of steel. We have seen that effeminate-looking man throttle a giant six feet four and broad in proportion, who was perfectly wild with the torture of the instrument and was struggling like a lion in the operating chair, wrench open his jaw and draw out the offending tooth as though he were handling an unruly child.

In one of the ante-rooms of the hall of meeting, there were a couple of those improved racks for torture called dentists chairs. Ah, how softly padded were those deceitful machines, and how bright the velvet with which they were covered. A red haired sanguine looking man was illustrating, with the help of an assistant, the vast advantages which they presented as compared with the old fashioned chair. That was a wonderful seat which the chief executioner was illustrating and expatiating upon with great volubility. It bristled with polished cranks and screws and slides that made our blood run cold as we saw them swiftly manipulated. A single turn of a crank would throw the unfortunate patient into a horizontal position and enable the operator to throttle him while thus helpless; another thrust and the chair swung to one side and the victim's head could be pinned under the operator's arm as in a vice; touching a lever and turning a crank shot the occupant several feet in the air, where he could be firmly held and his upper jaw devastated while he was helpless; touching the lever again and turning the crank lowered the sufferer to the floor in such a position that he could be secured motionless by the operator's knee. The eyes of those around us gleamed with strange satisfaction, and their countenances were lighted up with exultation as they watched these rapid movements. We alone stood spell-bound with horror. What if those savage men longing for a victum should seize upon us and pin us to that horrible rack? We fled from the spot.

As we passed up the room we saw another door ajar. There was no one within, but upon a long table were displayed all those keen and glittering instruments of torture found no where except in a dentist's operating room. A horrid fascination caused us to enter. Did you ever shudder at the dreadful display of tools known to no profession but that of the man who cures the tooth ache. We have, and those dreadful forms will live in memory while life shall last. Well, imagine all the blood chilling instruments of a score of dental establishments collected together and spread out in one awful array. There were big forceps and little forceps, cork screws and chisels, and big cant hooks, which looked as though they were made to lift the teeth of a mastidon. There were drills and punches, and bodkins, and files, and saws, and lancets, and screws, and sharp tubes, and dreadful looking machines for applying laughing gas or ether. To one side stood a bushel basket full of teeth, old roots, pieces of jaws on jaw, broken and forked fangs, decayed in all sorts of ways. They were grim trophies, and we retreated from this place even more speedily than from the first. If we don't have the night mare for a six months we shall consider ourselves lucky.

But now the hour of meeting had arrived, and the President of the Association, a veteran operator, with a countenance hard and stern, called to order.

The particular subject up for that day was operative dentistry. And presently there arose a sallow, feeble looking man, whose voice was also feeble and obstructed, to lecture. He first showed how the dentist could keep his accounts without having recourse to a book, and in a sort of dental short hand. Diagrams were used in the illustration and explanation, and we remember that the first was a female head, after some classical model perhaps, with the face cut up into four sections by lines passing, the one perpendicularly through the nose, and the other horizontally through the mouth. The next diagram showed the teeth as they would be divided by these lines, and we learned that we had thirty-two teeth, or at least should have that number, had not five or six of them retired from business. The four sections were named right superior, left superior, left inferior, and right inferior. The next cut showed four sets of figures, from one to eight, standing off like classes in a district school. These figures represented the teeth, and by an ingenious arrangement of hieroglyphics, the lecturer proposed to note at once what had beeu done to any particular tooth in the human head.

Now, said he, I regulate the amount of my charges just according to the amount of force, vitality or nervous energy expended in an operation. I will not work in any other way and my patients all understand it. I make my charges on the spot when I can calculate just how much vitality I have consumed in the work. The lecturer looked as though, on this scale the filling of a tooth would cost about $75 or $100.

The speaker now described the process of filling a tooth. It was very interesting. "Take," said he, "for instance, the second molar anterior, right proximo, cavity inclining towards the labial wall. First, cut away the face, smooth down, saw out and leave strong angles. Now commence with No. 0, put down the point firmly and strike with the lead mallet; build up all around, carefully filling the lower and upper inferior and superior arches and the right anterior cavity, using tools Nos. 1 and 2. Lay on the gold with the seratims and advance to the proper angles and finish off carefully the ridges and valleys, promontories and capes of the original.

This was clear and lucid, we began to feel like a dentist. The man is a wonderful one thought we. At the end of the lecture we found ourselves standing beside a tremendous six footer from

St. Louis. "Doctor," said we, "who is this doctor P—" (the lecturer.)

"He is," replied my gigantic friend, "a gentleman from Ohio; one of those erratic geniuses who are so difficult to follow. Now, with the exception of a few minor points, perhaps, in what does his mode of operation differ from yours?"

This was a stunner. Yes, to be sure—what was the difference. We were plunged into a profound reflection. We revolved the whole subject from beginning to end. We have thought of it constantly ever since, and we pledge you our word, reader, we can not up to this moment decide in what his mode of operation differs from ours. This is the penalty of being mistaken for a dentist. "In what ——," why, it may take years to solve that question."

---

